# Intra-Observer and Inter-Observer Reliability of Ankle Circumference Measurement in Patients with Diabetic Foot: A Prospective Observational Study

**DOI:** 10.3390/jcm12227166

**Published:** 2023-11-18

**Authors:** David Montoro-Cremades, Aroa Tardáguila-García, David Navarro-Pérez, Yolanda García-Álvarez, Mateo López-Moral, José Luis Lázaro-Martínez

**Affiliations:** 1 Diabetic Foot Unit, Clínica Universitaria de Podología, Facultad de Enfermería, Fisioterapia y Podología, Universidad Complutense de Madrid, 28040 Madrid, Spain; damontor@ucm.es (D.M.-C.); davinava@ucm.es (D.N.-P.); ygarci01@ucm.es (Y.G.-Á.); matlopez@ucm.es (M.L.-M.); diabetes@ucm.es (J.L.L.-M.); 2 Instituto de Investigación Sanitaria del Hospital Clínico San Carlos (IdISSC), 28040 Madrid, Spain

**Keywords:** diabetic foot, diabetic foot ulcers, oedema, ankle perimeter, ankle circumference, figure-of-eight method

## Abstract

Inflammation, being a typical response to vascular tissue alterations, induces variations in tissue oxygen diffusion pressure. Diabetic microangiopathy, an inflammatory process, is characterized by an increase in vascular flow at rest, reduced venous and arteriolar responses, and increased capillary permeability, resulting in oedema development, decreased transcutaneous oxygen pressure, and increased transcutaneous carbon dioxide pressure. This phenomenon potentially hampers ulcer healing. Although the figure-of-eight method has proven to be a reliable, valid, quick, and efficient test for assessing foot and ankle measurements in patients with oedema and compromised skin integrity, it has not been studied in patients with diabetic foot. The aim of this study was to determine and compare the intra- and inter-observer variabilities of the figure-of-eight method in patients with diabetic foot. A prospective observational and cross-sectional study was undertaken, involving sixty-one subjects from a specialized Diabetic Foot Unit. Three investigators with varying levels of experience independently measured the subjects to assess both intra-observer and inter-observer variability. The evaluation was conducted using the Intraclass Correlation Coefficient (ICC). In the statistical analysis, an ICC of 0.93, adjusted using a 95% confidence interval (CI), was obtained for inter-observer reliability ICC, indicating excellent reliability among observers. Furthermore, an ICC of 0.98 with a 95% CI was obtained for the intra-observer reliability analysis, indicating excellent reliability. The results support using this test during the clinical management of oedema in patients with diabetic foot. The absence of an objective, fast, and readily available diagnostic method for oedema in diabetic foot patients in clinical practice might pose a limitation. Subsequent research should tackle this issue and explore the correlation between ankle perimeter measurements and other clinical outcomes in diabetic foot patients, including wound healing and quality of life.

## 1. Introduction

Oedema is the perceptible increase in fluid volume within the interstitial space. It can be localized to a specific area of the body or result in generalized swelling. This process can occur because of a single local trigger or manifest as a clinical sign of a systemic complication or disorder [1].

Some of the most significant causes of oedema include several parameters: (1) increased capillary pressure, which can result from arteriolar vasodilation due to heat application or the presence of inflammation, or secondary to elevated venous pressure due to prolonged standing; (2) venous obstruction; (3) fluid retention during pregnancy or in premenstrual syndrome; (4) cardiac, renal, or hepatic insufficiency; (5) increased capillary permeability due to toxins, allergens, hypoxia, inflammation, or other causes; and (6) decreased plasma oncotic pressure due to nephrosis, starvation, or liver failure [1].

Among the previously mentioned risk factors for the development of oedema, heart failure and/or renal insufficiency (RI) are noteworthy. Diabetes mellitus (DM) stands out as the most prevalent cause of renal insufficiency globally. Cardiovascular disease (CVD) emerges as the leading cause of morbidity and mortality among individuals with DM, with RI significantly amplifying the risk of CVD [2]. Abnormal cardiac function often coexists with increased venous and capillary pressure, coupled with lower limb inflammation upon standing. The correlation between CVD and albuminuria is indicative of RI [3], aligning with the established association between oedema and CVD. This intricate web of connections underscores the multifaceted nature of these interrelated health conditions [4,5,6].

Diabetic foot ulcers (DFUs) are often associated with infection and inflammation, acting as local triggers that give rise to foot oedema. Inflammation, as a ubiquitous response to vascular tissue alterations, exhibits a discernible pattern in terms of changes in tissue oxygen diffusion pressure. Particularly noteworthy is the vasodilation phase, during which oxygen pressure experiences a slight increase, followed by a subsequent decline correlated with the onset of oedema and the subsequent elevation in hydrostatic pressure. This intricate interplay has the potential to impede the healing process of DFUs, emphasizing the intricate dynamics underlying diabetic foot complications [7,8].

The proper supply and control of oxygen to a specific region depends on numerous factors, including capillary density, local flow rate, blood viscosity, and hemoglobin release pressure. The efficiency of oxygen diffusion through different tissues depends on the nature of the intercellular matrix, cellular density under physiological or pathological conditions, and whether the intercellular space is affected by oedema, the presence of microorganisms, and/or devitalized tissue. Hemostatic pressure (HP) in capillaries can be influenced by any external pressure above it, resulting in vessel compression. An increase in intrinsic pressure can occur due to the presence of oedema. If this pressure cannot be released, it will result in capillary collapse and tissue hypoxia [7,8].

Furthermore, additional diabetes-related processes play a pivotal role in the manifestation of this phenomenon. Diabetic microangiopathy, a distinctive feature of diabetes, is marked by increased vascular flow at rest, diminished responses in venous and arteriolar functions, and elevated capillary permeability. These intricate vascular changes collectively contribute to the onset of oedema, a decline in transcutaneous oxygen pressure, and an escalation in transcutaneous carbon dioxide pressure [7,8]. Understanding the multifaceted impact of diabetes-related alterations is crucial for comprehending the complex pathophysiology underlying diabetic foot complications.

The comprehensive assessment of diabetic foot ulcers (DFUs) requires thorough examinations of vascular and neurological functions, coupled with a meticulous evaluation of ulcer characteristics. Successful treatment strategies predominantly include offloading utilizing suitable devices and prompt management of concurrent infections through procedures like debridement and, when warranted, revascularization [7,8]. Despite the acknowledged detrimental impact of oedema on the healing process [9], the current clinical management protocols for lesions do not explicitly incorporate oedema-targeted interventions [10]. Recognizing the significance of addressing oedema within the overall management framework may unveil novel avenues for optimizing DFU treatment outcomes.

The measurement of inflammation serves as a crucial tool for clinicians, enabling the evaluation of inflammation severity, formulation of treatment strategies, and assessment of treatment efficacy based on objective observations, rather than relying solely on subjective criteria. In this context, it becomes imperative to meticulously evaluate the accuracy and consistency of tests employed for determining foot and ankle measurements in patients with oedema [9,10]. This analysis is pivotal for ensuring the reliability of clinical assessments and enhancing the precision of treatment interventions.

Water displacement volume, along with ankle circumference measurements using the Figure-of-Eight method, stand out as the most practical and reliable approaches for quantifying ankle oedema [11,12]. While volumetry is traditionally considered the gold standard, its application is cautioned in patients with open ulcers or those in the early post-operative stages [13,14]. This emphasizes the importance of employing alternative, yet equally dependable, methods like the Figure-of-Eight method for comprehensive oedema assessment in a diverse patient population.

The figure-of-eight method, a non-invasive and cost-effective test, offers an objective quantification of ankle oedema through the utilization of a tape measure and specific bony landmarks around the ankle joint [15]. Demonstrating reliability (intraclass correlation coefficient (ICC) 0.94) and validity (r = 0.65; *p* < 0.001), it stands as a swift and efficient alternative (*p* < 0.001) to volumetry for evaluating foot and ankle measurements in patients with oedema and compromised skin integrity [16]. Despite its established merits, it is crucial to note that this test has not undergone exploration in the context of diabetic foot patients.

Given the potential benefits of implementing a reliable, reproducible, non-invasive, and cost-effective method for objective oedema quantification in patients with DFUs—crucial for assessing severity and formulating treatment strategies in clinical practice—this study aimed to determine and compare intra- and inter-observer variabilities using the figure-of-eight method in individuals with diabetic foot.

## 2. Materials and Methods

### 2.1. Study Design

The study’s design involves observing and documenting individual measurements over time, without intervening in the natural progression of the pathology. A prospective observational and cross-sectional study was conducted following the guidelines outlined in Strengthening the Reporting of Observational Studies in Epidemiology (STROBE) [17].

During a single visit per patient between November 2022 and March 2023, measurements were conducted by three investigators in a specialized Diabetic Foot Unit. As a prospective observational and cross-sectional study, no follow-up was necessary. The investigators, with varying levels of experience, independently measured ankle circumferences. One investigator had five years of experience in ankle circumference measurement, another had one year of experience, and the third had no prior experience but was trained before the start of the study. To mitigate observer bias, the investigators measured ankle circumferences on the same subjects without knowledge of each other’s measurements.

Reliability pertains to the reproducibility of results obtained through a measurement procedure. Intra-observer reliability gauges the agreement when a measurement is repeated by the same observer under identical conditions. Inter-observer reliability assesses the agreement when a measurement is repeated under identical conditions by different observers. Lack of reliability may stem from discrepancies in measurement instruments or the instability of the attribute being measured. Intra- and inter-observer variabilities were evaluated using the Intraclass Correlation Index (ICC) with a 95% confidence interval (CI).

Study participants, aged 18 and above, were eligible if diagnosed with type 1 or type 2 diabetes mellitus, had a history of previous ulceration, and had signed the informed consent form. Exclusion criteria included patients with active ulcers and/or diagnosed with rheumatoid arthritis, as these may introduce potential bias due to non-diabetes-related ankle inflammation.

### 2.2. Study Variables

The dependent variables of the study were the ankle circumference measurements taken by the three observers. The independent variables included demographic variables, such as age (years); gender (male/female); weight (kg); height (cm); body mass index (BMI); DM diagnosis (type 1, type 2 insulin-dependent, type 2 non-insulin-dependent); duration of DM (years); presence of peripheral vascular disease diagnosed via the presence or absence of distal pulses; ankle-brachial index (ABI) or toe-brachial index (TBI); presence of diabetic neuropathy diagnosed using the Semmes–Weinstein Monofilament (5.07 mm–10 g) for sensory testing and a biothesiometer (>25 mV) for vibratory sensibility assessment; and a personal medical history of smoking, hypercholesterolemia, diabetic nephropathy, cardiovascular conditions, venous insufficiency, Charcot neuroarthropathy, oedema, aetiology of oedema, and/or fractures.

### 2.3. Procedure

**Figure 1:**
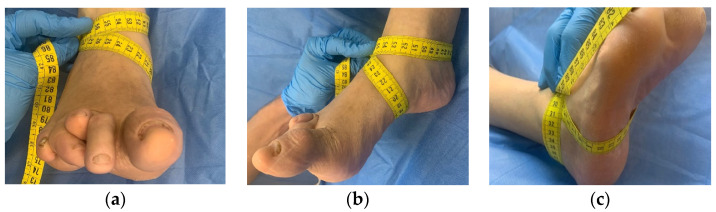
Ankle circumference measurement procedure. (**a**) For the measurement of the subtalar joint, the starting point of the tape measure was placed at the midpoint between the tendon of the anterior tibialis muscle and the lateral malleolus. (**b**) The tape was then moved medially, until it reached the distal limit of the navicular tuberosity, continued along the medial arch, traversed the plantar surface towards the proximal limit of the base of the styloid process of the fifth metatarsal, and crossed over the anterior tibialis tendon. (**c**) The tape measure continued around the ankle joint with the tape positioned distal to the apex of the medial malleolus so that it crossed the Achilles tendon on the posterior surface and traversed the lateral aspect of the limb through placing the tape below the apex of the lateral malleolus with the end of the measurement at the starting point of the tape.

The ankle circumference measurement was carried out according to the original description of the test [15] using a tape measure to measure inflammation at the talar and subtalar joint level, forming a figure resembling the shape of an “8” (Figure 1).

The measurement was taken with subjects in a seated position with extended legs using a tape measure that was calibrated in centimeters. The ankle joint was held in a neutral position to eversion and inversion while maintaining a 90° ankle flexion position, if the subject’s range of motion allowed.

The reference points considered for the measurement included several anatomical parameters: (1) tuberosity of the navicular bone, (2) base of the styloid process of the fifth metatarsal, (3) distal boundary of the medial malleolus, (4) distal boundary of the lateral malleolus, and (5) tendon of the anterior tibialis muscle.

For the measurement of the subtalar joint, the starting point of the tape measure was placed at the midpoint between the tendon of the anterior tibialis muscle and the lateral malleolus. The tape was then moved medially, until it reached the distal limit of the navicular tuberosity, continued along the medial arch, traversed the plantar surface towards the proximal limit of the base of the styloid process of the fifth metatarsal, and crossed over the anterior tibialis tendon. Subsequently, for the measurement of the talar joint, the tape measure continued around the ankle joint with the tape positioned distal to the apex of the medial malleolus so that it crossed the Achilles tendon on the posterior surface and traversed the lateral aspect of the limb through placing the tape below the apex of the lateral malleolus with the end of the measurement at the starting point of the tape.

### 2.4. Risk of Bias

Consideration of potential biases inherent in observational studies is crucial, and the implementation of effective strategies to prevent these biases is equally important for ensuring the study’s integrity and reliability. Addressing these risks enhances the internal validity of the research, providing a more robust foundation for drawing meaningful conclusions from the observational data. Through acknowledging and managing potential biases, the study aims to uphold the highest standards of scientific rigor and contribute to the overall credibility of the findings.

Concerning internal validity, a proactive approach was taken to mitigate potential selection bias through implementing a prospective study design. This strategic choice was made to ensure that the process of subject recruitment and selection occurred prior to the event of interest. Through adopting this method, the study aims to minimize the likelihood of obtaining inaccurate or skewed results related to the primary objectives. This careful consideration of study design enhances the internal validity, reinforcing the reliability and accuracy of the research outcomes.

To minimize the risk of information or measurement bias, a standardized approach was employed. All observers consistently utilized the same measuring instrument throughout the study, effectively reducing the likelihood of differential error. This attention to maintaining uniformity in measurement tools contributes to ensuring a consistent and reliable methodology across all observations.

To mitigate potential biases during the planning phase of the study, a comprehensive strategy was implemented. This involved thorough training for all observers, equipping them with the necessary skills and knowledge essential for precise measurements. Additionally, blinding procedures were rigorously enforced during the measurement process, ensuring that observers remained impartial and unbiased. This multifaceted approach to study planning safeguards against potential sources of bias, enhancing the credibility and reliability of the study’s outcomes.

To minimize the potential impact of memory and data collection bias, a meticulous approach was employed. Information furnished by the participants underwent thorough cross-verification, aligning with the reports obtained under their prior consent. This dual-check mechanism ensured the accuracy and reliability of the collected data. Furthermore, the data collection instrument was deliberately designed with simplicity, featuring dichotomous response options and the recording of quantitative variables. This comprehensive strategy not only bolsters the integrity of the information gathered but also strengthens the study’s overall methodological robustness.

Strategic measures were meticulously implemented to mitigate potential bias during the analysis and interpretation of results. To strengthen the methodological validity, the study adhered to the guidance provided by Portney and Watkins [18]. This involved an accurate selection of statistical tests, ensuring their appropriateness, and a meticulous approach to result interpretation. Through conscientiously following established guidelines, the study aimed to enhance the reliability and objectivity of the data analysis, contributing to the strength of the overall research findings.

No specific interventions were considered imperative to address potential confounding bias, given its intrinsic control within the study’s case-control design. Correspondingly, preventive measures to avert loss to follow-up were deemed unnecessary, aligning with the study’s nature as a prospective cross-sectional observational inquiry that did not necessitate subsequent follow-up assessments. The study design inherently encapsulated elements to safeguard against confounding variables and precluded the need for additional strategies to mitigate loss to follow-up, reinforcing the study’s methodological coherence.

To mitigate biases influencing external validity, careful consideration was given to the time necessary for measuring the outcome variable. This was achieved through employing a technique renowned for its ease of reproducibility in a clinical setting and its cost-effectiveness, ultimately enhancing the generalizability of the study’s findings.

### 2.5. Statistical Analysis

Statistical analysis was performed using the IBM SPSS^®^ Statistics 27 software (IBM, Armonk, NY, USA). Categorical variables were analysed in terms of frequencies and percentages. The analysis of quantitative variables involved calculating the mean and standard deviation.

Before conducting hypothesis tests, the normality of quantitative variables was assessed using the Kolmogorov–Smirnov test to verify their distribution. To measure overall agreement between two or more measurements involving quantitative variables, intra- and inter-observer variabilities were evaluated using the two-way random effects, absolute agreement, single rater/measurement ICC (2,1) with a 95% CI. Statistically significant values were considered for *p* < 0.05 with a 95% CI, and values of β were established to achieve a study power of 80%. Interpretation of the ICC values followed the guidelines of Portney and Watkins [18] in which an ICC value < 0.5 indicates poor reliability, a value between 0.51 and 0.75 indicates moderate reliability, and a value > 0.90 indicates excellent reliability. The standard error (SE) was calculated to interpret the inherent measurement error.

### 2.6. Ethical Principles

The study adhered to the ethical principles outlined in the Declaration of Helsinki [19] and complied with the prevailing national legislation governing research involving patients as research subjects. Approval for the study protocol, with internal code C.I. 22/612-E, was obtained from the Research Ethics Committee of Hospital Clínico San Carlos.

Prior to inclusion, participants received detailed information about the study and its procedures. Subsequently, they expressed voluntary agreement to participate through informed consent for the collection of data for scientific purposes.

To uphold data confidentiality, the data collection notebook maintained anonymity and the study results were securely stored in dedicated files created specifically for this purpose, following the security measures mandated by current legislation in Spain.

## 3. Results

### 3.1. Clinical Characteristics of Patients

Sixty-one Caucasian subjects participated in the study (Table 1) with forty-two (69%) males and nineteen (31%) females. The mean age was 69.6 ± 9.7 years. No missing data for any variables of interest were found. The mean body mass index (BMI) was 28 ± 5 (27.7 ± 5.6 for males and 28.4 ± 3.34 for females). In our study, the most prevalent type of diabetes was type 2, with fifty-three subjects (87%), compared to type 1 diabetes with eight subjects (13%). Fifteen subjects (26%) had a diagnosis of Peripheral Arterial Disease (PAD), and twenty-five subjects (41%) had diabetic neuropathy (DN). Twenty-seven subjects (44%) presented clinical signs of lower limb oedema, with the most frequent recorded aetiology being venous insufficiency (36.1%) followed by trauma (3.3%) or Charcot neuroarthropathy (4.9%).

### 3.2. Reliability Analysis

#### 3.2.1. Inter-Observer Reliability Analysis

In the reliability analysis, despite the measurement means (Table 2) being higher for the experienced observer, the ICC adjusted with a 95% CI presented a value of 0.93 (0.89–0.95; *p* < 0.0001), indicating excellent reliability among observers (Table 3). Correlations of 0.95 between the experienced and moderately experienced observers, 0.93 between the moderately experienced and inexperienced observers, and 0.91 between the experienced and inexperienced observers were found (Table 4).

#### 3.2.2. Intra-Observer Reliability Analysis

In the intra-observer reliability analysis (Table 5), an ICC of 0.98 (0.98–0.99; *p* < 0.0001) with a 95% CI was obtained. The correlation between elements as 0.98 between the first measurement by the experienced investigator and the second measurement, 0.99 between the second observation and the third, and 0.98 between the first and third measurements, indicating excellent intra-observer reliability.

## 4. Discussion

The comprehensive evaluation of cases and continuous assessment of changes throughout the management of pathologies are essential to accurately gauge their severity and ascertain the effectiveness of the proposed treatment. In this context, maintaining a meticulous approach to minimize errors in the measurement of oedema becomes crucial. This meticulousness not only enhances the reliability of assessments but also ensures consistency, facilitating a more accurate interpretation of results over the course of the disease management process.

While the figure-of-eight method has been acknowledged for its objectivity, reliability, validity, and efficiency, serving as a promising alternative to volumetry in assessing oedema in the foot and ankle, a notable research gap exists concerning its application in individuals with diabetic foot conditions. Despite its recognized utility, there is a scarcity of published studies examining its relevance in the context of diabetic foot cases. Thus, the primary aim of this study was to systematically investigate and compare both intra- and inter-observer variabilities of the figure-of-eight method specifically in patients with diabetic foot.

The measurement technique was systematically implemented by three distinct observers on individuals with diabetic foot conditions, followed by rigorous statistical analysis. As per the result analysis guidelines outlined by Portney and Watkins [18], the inter-observer reliability yielded an ICC value of 0.93 (0.89–0.95; *p* < 0.0001) with a 95% CI, signifying excellent consistency among the observers. Furthermore, the intra-observer reliability exhibited an ICC value of 0.98 (0.98–0.99; *p* < 0.0001) with a 95% CI, affirming excellent reliability within each observer’s measurements.

The findings of the current study align with outcomes reported in investigations conducted by Rohner-Spengler et al. [13], wherein measurements on individuals with oedema linked to ankle fractures demonstrated an ICC ranging from 0.98 to 0.99 with a 95% CI (*p* < 0.001). Similarly, Devoogdt et al. [16] observed comparable results when applying measurements on individuals with lymphoedema, yielding an ICC of 0.85 to 0.98 with a 95% CI (*p* < 0.001).

The measurement of ankle circumference has been previously employed as a tool in other research and for monitoring in clinical practice related to various medical conditions. For instance, Guex et al. [20] utilized this technique to assess correlations between ankle circumference, symptoms, and quality of life in lower limb oedema. In a recent randomized clinical trial, Cacchio et al. [21] employed this measurement to monitor the progression of oedema reduction in post-traumatic patients treated with diosmin, coumarin, and arbutin compared to conventional treatment. Another randomized clinical trial utilized ankle circumference measurement to compare the effectiveness of compression stockings in preventing lower limb oedema and its association with comfort after three hours of flight [22].

The guidelines for diagnosing and treating active Charcot neuro-osteoarthropathy (CN) in individuals with diabetes, as proposed by the International Working Group on the Diabetic Foot, designate unilateral oedema as a clinical indicator of active CN. They propose that monitoring the oedema could aid in identifying the remission phase of the condition. However, there is insufficient evidence to substantiate the use of reliable techniques for such monitoring or to establish a correlation between their results and radiological findings. Consequently, the management of CN is currently restricted to the subjective quantification and diagnosis of oedema [23].

Our findings, indicating favorable intra- and inter-observer reliability, support the use of this assessment test in clinically managing oedema in diabetic foot patients. This test could aid clinicians in detecting and managing complications like Charcot neuro-osteoarthropathy and serve as a valuable tool for future investigations. Notably, it is an easily applicable, cost-effective, and reproducible test, requiring only basic knowledge of lower limb anatomy and a short training period in the measurement technique.

Strengths of this study include being the first to correlate ankle perimeter measurements with oedema in diabetic foot patients. However, limitations exist as no studies explaining the pathophysiology or prevalence of oedema specifically in diabetic foot patients were found. This limitation affects sample size calculations, leading to sampling bias and affecting the generalizability of results. The absence of a quick and accessible diagnostic method for oedema in diabetic foot patients complicates clinical practice, which relies on subjective clinical signs [11,12,24]. This may introduce selection bias. Additionally, no studies were found comparing ankle perimeter measurements in diabetic foot patients with those in individuals with normal feet or assessing the reliability of these measurements in the presence or absence of oedema, potentially introducing observer differences.

Future research should address these gaps, exploring the relationship between ankle perimeter measurements and various clinical outcomes in diabetic foot patients, such as wound healing and quality of life.

## 5. Conclusions

In summary, the figure-of-eight method exhibited excellent inter- and intra-observer reliability in patients with diabetic foot. Integrating this assessment test into clinical practice for oedema management in diabetic foot patients could aid clinicians in detecting and addressing complications, while also providing a valuable tool for future research endeavors.

## Figures and Tables

**Table 1:** Demographic characteristics of the sample.

Variable	Mean	Standard Deviation
Age (years)	69.7	9.8
DM Evolution (years)	18.2	10.1
Height (cm)	169.8	11.3
Weight (kg)	81.2	20.8
BMI	28.0	5.0

Abbreviations: DM, diabetes mellitus; BMI, body mass index. Sixty-one individuals diagnosed with type 1 or type 2 diabetes mellitus, aged 18 or older and with a non-active history, were consecutively enrolled in the study.

**Table 2:** Differences between means of the observers’ measurements.

Observer	Mean	Standard Deviation
First observer ^1^	55.5	3.5
Second observer ^2^	54.4	3.5
Third observer ^3^	55.2	3.8

^1^ First observer: investigator with five years of experience in ankle circumference measurement. ^2^ Second observer: investigator with one year of experience in ankle circumference measurement. ^3^ Third observer: investigator with no prior experience but received training before the study commencement.

**Table 3:** Intraclass correlation coefficient about inter-observer reliability.

Measurements ^1^	Intraclass Correlation ^2^	95% Confidence Interval	Sig
Single measurements	0.93	0.89–0.95	<0.001
Mean measurements	0.96	0.96–0.98	<0.001

^1^ Each observer performed three measurements for each subject in the study. The “single measurements” row denotes the comparison between observers for each measurement, while the “mean measurements” row represents the difference between the means of the measurements by the observers. ^2^ This table illustrates the agreement between measurements by each observer. According to Portney and Watkins’ guidelines [18], the ICC values obtained suggest excellent inter-observer reliability.

**Table 4:** Correlation matrix between inter-observer reliability elements.

Observer	First Observer ^1^	Second Observer ^2^	Third Observer ^3^
First observer ^1^	1	0.95	0.91
Second observer ^2^	0.95	1	0.93
Third observer ^3^	0.91	0.93	1

This table illustrates the agreement between measurements by each observer. As per Portney and Watkins’ guidelines [18], the ICC values obtained suggest excellent inter-observer reliability. ^1^ First observer: investigator with five years of experience in ankle circumference measurement. ^2^ Second observer: investigator with one year of experience in ankle circumference measurement. ^3^ Third observer: investigator with no prior experience but received training before the study commencement.

**Table 5:** Intraclass correlation coefficient of intra-observer reliability.

Measurements	Intraclass Correlation ^3^	95% Confidence Interval	Sig
Single measurements ^1^	0.98	0.98–0.99	<0.001
Mean measurements ^2^	0.99	0.99–0.99	<0.001

This table displays the results obtained from comparing the measurements made by each individual observer. ^1^ The “Single measurements” row denotes the comparison of measurements taken by each observer. ^2^ The “Mean measurements” row represents the comparison between the means of the measurements taken by each observer. ^3^ According to the guidelines by Portney and Watkins [18], the ICC values obtained indicate excellent intra-observer reliability.

## Data Availability

Data are contained within the article and supplementary materials.
